# Labor, Puerperal Fever, and Death

**Published:** 1874-03

**Authors:** Geo. J. Monroe

**Affiliations:** Leland, Ill.


ARTICLE III.—*Labor, Puerperal Fever, and Death.* By GEO. J. MONROE, M.D., Leland, Ill.

Mrs. Catharine Apt; German; aged 20; primapara; taken in labor December 22d, 1873. Mrs. Apt was very fleshy; adipose tissue abundant. When pregnant about two months she met with a fall, fracturing the leg in the lower third. After recovering from this injury she was pretty well for the balance of the time of her pregnancy, with the exception of persistent constipation, frequently going five or six days without an evacuation of the bowels; still, no great inconvenience resulted therefrom.

At about six o'clock P. M., December 22d, while at the supper table, the membranes ruptured without any premonitory symptoms, allowing the liquor amnii to escape. After the escape of the waters a severe hemorrhage commenced. Her husband got her to bed as soon as he could, called in a neighboring woman, and sent for me as speedily as possible.

I got there about half past eight, having seven miles to go. She was still flowing profusely; the blood had saturated two quilts, two woolen skirts, one woolen shirt, and a piece of rag carpet; still, she did not seem to be much prostrated. I at once gave her two teaspoonfuls of Tilden's fluid extract of ergota; made a digital examination; found the womb soft and flabby; os dilated so I could introduce two fingers; head presentation. As yet she had had no pains. In about fifteen minutes after administering the ergota the hemorrhage ceased, and did not again appear to any amount until after the expulsion of the child. She had no pain during the night or next day until about six P. M., at which time labor pains commenced, and went on naturally until about three A. M. of the 24th, when she was delivered of a puny, feeble girl. The cord was twenty-two inches long, and about the size of fence wire. I have never seen so small a cord during my practice of thirteen years. After the child was born, and on making slight traction on the cord, it at once separated from the placenta—something that never happened with me before. I waited one hour for the expulsion of the after-birth, but it did not come; neither was there the slightest indication of pain. Gave two teaspoonfuls of ergota fluid extract. Waited one half hour—still no pain. Introduced my hand to deliver placenta; on reaching the os, I found it so much contracted that it required some effort to introduce the index finger. Made forcible dilatation, and reached the placenta, which was high up in the uterus; had to use my fingers to detach a portion of it from the womb; finally succeeded in delivering it.

After the removal of the placenta there was but little flowing; the womb seemed to contract naturally. After remaining about one hour, everything being apparently right, and the lady very comfortable, I left.

She remained comfortable until about six P. M., Wednesday evening, when all at once she was taken with severe uterine pains; several large coagula of blood were expelled.

I was sent for. On my arrival at 9 P. M. I found her suffering as much as she had at any time during her labor. On examination found no coagula in the womb; uterus very hot to the feel, and unusually dry. Gave opii in full doses to control the pain, and ordered castor oil to move the bowels.

Was sent for early Thursday morning. Pain still continued; no movement of the bowels; pulse 140; profuse perspiration. Puerperal fever, well marked. Gave morph. sulphas, gr. j, every three hours; ordered calomel, gr. xv, to be followed in three hours by castor oil, oz. ss., spirits turpentine, gtts. xxx; this to be followed in ten hours, if no operation, by injections of warm water.

Eight P. M. Thursday: abdomen tympanitic; bowels had moved two or three times; pain less severe; no sleep; pulse 140. Continue morphine, with quinine, gr. ij, with each powder. Turpentine over bowels. Egg-nog and beef tea.

Friday morning I found that she had vomited a great deal during the night. Pain severe; lochia had ceased; bowels had moved twice; great tympanitis; considerable flatulence; urinates freely; some hiccough; pulse 130. Ordered gr. j sulph. morph. every hour until rest was obtained. Nourishment and stimulants if the stomach will retain them.

Friday evening: had rested a good deal during the day; pulse 145; had taken some nourishment. Continue morphine sufficiently often to control pain; quinine, nourishment and stimulants; warm application over abdomen.

Saturday morning: had slept some during the night; bowels had moved twice; pulse 140, very feeble; hiccough produced by any movement; wanted beer. Continue treatment; give beer in limited quantities.

Sunday morning: stomach rejects everything taken; hiccough almost continually; eyes very much injected; some difficulty in vision—in trying to reach a raveling on the bed, fell short about an inch with each effort. Death imminent. Use morphine and quinine dissolved in beef tea, by injections per rectum. Purulent and very offensive discharges from vagina. Ordered injections of warm water and carbolic acid per vaginam.

Monday morning: eyes more injected; rested some during the night; had taken a good deal of beer; pulse rapid and very feeble; respiration labored; tympanitis great; feet cold and clammy; anxious expression; contracted features; mind clear; abdomen purple. Continue injections.

On my arrival Tuesday morning at 10 A. M. she had just breathed her last. Did not rest any during the night; suffered a great deal of pain; vomiting and hiccough all night. Retained her senses until the last moment.

The questions that arise in my mind, are the following: What was the cause of the hemorrhage before labor? had the fall in the early stages of pregnancy anything to do with it? Why was the cord so unusually small? why did it separate so easily from the placenta? Might not the remaining quiet during the treatment of the fractured leg have produced the persistent constipation? Is puerperal fever any more apt to occur where we have hemorrhage than when we do not?

I attended four other ladies during the same week; no unusual symptoms present in either of the cases; convalescence natural in all.

